# Whither systems medicine?

**DOI:** 10.1038/emm.2017.290

**Published:** 2018-03-02

**Authors:** Rolf Apweiler, Tim Beissbarth, Michael R Berthold, Nils Blüthgen, Yvonne Burmeister, Olaf Dammann, Andreas Deutsch, Friedrich Feuerhake, Andre Franke, Jan Hasenauer, Steve Hoffmann, Thomas Höfer, Peter LM Jansen, Lars Kaderali, Ursula Klingmüller, Ina Koch, Oliver Kohlbacher, Lars Kuepfer, Frank Lammert, Dieter Maier, Nico Pfeifer, Nicole Radde, Markus Rehm, Ingo Roeder, Julio Saez-Rodriguez, Ulrich Sax, Bernd Schmeck, Andreas Schuppert, Bernd Seilheimer, Fabian J Theis, Julio Vera, Olaf Wolkenhauer

**Affiliations:** 1 European Molecular Biology Laboratory, European Bioinformatics Institute (EMBL-EBI), Wellcome Genome Campus, Hinxton, Cambridge, UK; 2 Institute of Medical Statistics, University Medical Center Göttingen, Göttingen, Germany; 3 Fachbereich Informatik und Informationswissenschaft, Universität Konstanz, Konstanz, Germany; 4 Institute of Pathology, Charité Universitätsmedizin, Berlin, Germany; 5 Integrative Research Institute for the Life Sciences, Institute for Theoretical Biology, Humboldt Universität, Berlin, Germany; 6 Biologische Heilmittel Heel GmbH, Baden-Baden, Germany; 7 Department of Public Health and Community Medicine, Tufts University School of Medicine, Boston, MA, USA; 8 Perinatal Epidemiology Unit, Hannover Medical School, Hannover, Germany; 9 Center for Information Services and High Performance Computing, Technische Universität Dresden, Dresden, Germany; 10 Institute for Pathology, Neuropathology, Hannover Medical School, Hannover, Germany; 11 Institute for Neuropathology, University Clinic of Freiburg, Freiburg, Germany; 12 Institute of Clinical Molecular Biology, Christian-Albrechts-University of Kiel, Kiel, Germany; 13 Institute of Computational Biology, Helmholtz Zentrum München, Neuherberg, Germany; 14 Center for Mathematics, Technische Universität München, Garching, Germany; 15 Transcriptome Bioinformatics, LIFE Research Center for Civilization Diseases, University of Leipzig, Leipzig, Germany; 16 Division of Theoretical Systems Biology, German Cancer Research Center (DKFZ), Heidelberg, Germany; 17 BioQuant Center, University of Heidelberg, Heidelberg, Germany; 18 Maastricht Center for Systems Biology, University of Maastricht, Maastricht, The Netherlands; 19 Institute of Bioinformatics, University Medicine Greifswald, Greifswald, Germany; 20 Division of Systems Biology of Signal Transduction, German Cancer Research Center (DKFZ), Heidelberg, Germany; 21 Translational Lung Research Center (TLRC), Member of the German Center for Lung Research (DZL), Heidelberg, Germany; 22 Molecular Bioinformatics, Institute of Computer Science, Johann Wolfgang Goethe-University Frankfurt am Main, Frankfurt am Main, Germany; 23 Center for Bioinformatics, University of Tübingen, Tübingen, Germany; 24 Quantitative Biology Center, University of Tübingen, Tübingen, Germany; 25 Biomolecular Interactions, Max Planck Institute for Developmental Biology, Tübingen, Germany; 26 Computational Systems Biology, Bayer Technology Services GmbH, Leverkusen, Germany; 27 Department of Medicine II, Saarland University Medical Center, Saarland University, Homburg, Germany; 28 Biomax Informatics AG, Planegg (Munich), Germany; 29 Department of Computational Biology and Applied Algorithmics, Max-Planck-Institut für Informatik, Saarbrücken, Germany; 30 Institute for Systems Theory and Automatic Control, University of Stuttgart, Stuttgart, Germany; 31 Stuttgart Research Center Systems Biology, University of Stuttgart, Stuttgart, Germany; 32 Department of Physiology & Medical Physics, Centre for Systems Medicine, Royal College of Surgeons in Ireland, Dublin 2, Ireland; 33 Institute of Cell Biology and Immunology, University of Stuttgart, Stuttgart, Germany; 34 Institute for Medical Informatics and Biometry, Carl Gustav Carus Faculty of Medicine,Technische Universität Dresden, Dresden, Germany; 35 Joint Research Centre for Computational Biomedicine (JRC-COMBINE), RWTH-Aachen University Hospital, Aachen, Germany; 36 Department of Medical Informatics, University Medical Center, Göttingen, Germany; 37 Institute for Lung Research/iLung, Universities of Giessen and Marburg Lung Center, Philipps University Marburg, Marburg, Germany, Member of the German Center for Lung Research, Marburg, Germany; 38 Joint Research Center for Computational Biomedicine, AICES, RWTH Aachen University, Aachen, Germany; 39 Department of Mathematics, Technische Universität München, Munich, Germany; 40 Laboratory of Systems Tumor Immunology, Friedrich-Alexander University Erlangen-Nürnberg and Erlangen University Hospital, Erlangen, Germany; 41 Department of Systems Biology and Bioinformatics, Rostock University, Rostock, Germany; 42 Stellenbosch Institute for Advanced Study (STIAS), Wallenberg Research Centre at Stellenbosch University, Stellenbosch, South Africa

## Abstract

New technologies to generate, store and retrieve medical and research data are inducing a rapid change in clinical and translational research and health care. Systems medicine is the interdisciplinary approach wherein physicians and clinical investigators team up with experts from biology, biostatistics, informatics, mathematics and computational modeling to develop methods to use new and stored data to the benefit of the patient. We here provide a critical assessment of the opportunities and challenges arising out of systems approaches in medicine and from this provide a definition of what systems medicine entails. Based on our analysis of current developments in medicine and healthcare and associated research needs, we emphasize the role of systems medicine as a multilevel and multidisciplinary methodological framework for informed data acquisition and interdisciplinary data analysis to extract previously inaccessible knowledge for the benefit of patients.

## What is in the name ‘Systems Medicine’?

Personalized medicine, precision medicine, P4 medicine (P4=predictive, preventive, personalized and participatory) and systems medicine are different names to illustrate the common desire to establish a novel (more personalized, precise and systematic) approach in medicine.^1^ Despite the different names, the corresponding disciplines share goals of improved diagnosis, targeted therapy, better prognosis and prevention. The routes by which these goals should be achieved are very similar and a core element is the integration of data from different sources, including conventional patient data, clinicopathological parameters, molecular and genetic data as well as data generated by additional new-omics technologies. It is evident that this endeavor will require investigator teams combining the expertise from different disciplines.^2, 3, 4^

To promote ‘systems medicine’ over the other terms, one could argue that medicine has always been ‘personalized’ any diagnosis, therapy and prognosis is always about a particular individual, taking into account the patient's specific condition. Whether medicine, medical diagnosis and (pharmaceutical) treatment will ever reach the level of precision we aim and hope for can be debated. However, it appears that systems medicine indeed differs substantially from traditional medical approaches.^2, 3, 4^

## Why ‘Systems’?

A system is formally and intuitively a set of related objects. In biomedicine, systems are for example networks of interacting molecules and populations of interacting cells. The first step in a systems approach is to identify the system variables that are relevant for a particular question at hand.

The key system variables are those that will be measured and whose observations will be used to answer a question. In the next step, the interactions of these variables are characterized. At the molecular level, this could be the identification of the molecules acting on a particular receptor. At the cellular level this could be the identification of cell types involved in a particular disease. At the level of the whole body one would discuss the interaction of physiological parameters. How interactions at the molecular, cellular or physiological level are characterized, defined or hypothesized, is a central question of ‘modeling’, the process by which a complex system is investigated through a reduced representation.

The final step in the systems approach is to investigate the consequences of particular interactions, the emergent patterns or behavior of the system (induced by the interaction of the system’s entities).^5^ The systems approach is a systematic approach to identify the elements that contribute to the understanding of an actual situation and specific problem. For this purpose, suitable approaches must be designed to generate data that can parameterize and validate a model and predict the behavior of a system. Each step of such a systems approach is supported by statistical, mathematical and computational tools. The long tradition of systems approaches in the physical and engineering sciences, as well as more recent developments in systems biology will benefit systems medicine.

A landmark example of how differential equation models can cause paradigm shifts in disease treatment is the discovery of the high HIV turnover using dynamical systems models that led to modern combinatorial treatments.^6^ However, systems approaches are not limited to the dynamical systems theory and differential equation models,^7, 8^ and current approaches often combine a range of statistical, mathematical and computational approaches in workflows to analyze and interpret data.^9^ But also abstract models can challenge clinical practice, and are thus of direct relevance to medical practice.^10^ While the classical bio-statistical analysis has a long history and its importance is widely accepted in evidence-based medicine, systems medicine requires a wider range of mathematical and computational techniques. Heterogeneity of data is becoming a key challenge in medical research, which implies that the analysis of the data becomes more complex and the range of methodologies that are required is widening. The analysis of medical data increasingly involves diverse computational and mathematical expertise, ranging from statistical approaches to machine learning, and involving diverse modeling paradigms such as rate equations, logical models or agent-based simulations.^11, 12, 13, 14^ Although new IT infrastructure is clearly a prerequisite of systems approaches, digitalization of medicine will require expanding the multi-disciplinary expertise in these diverse fields of computational and mathematical research.

## Integrated systems medicine workflows

**Figure 1:**
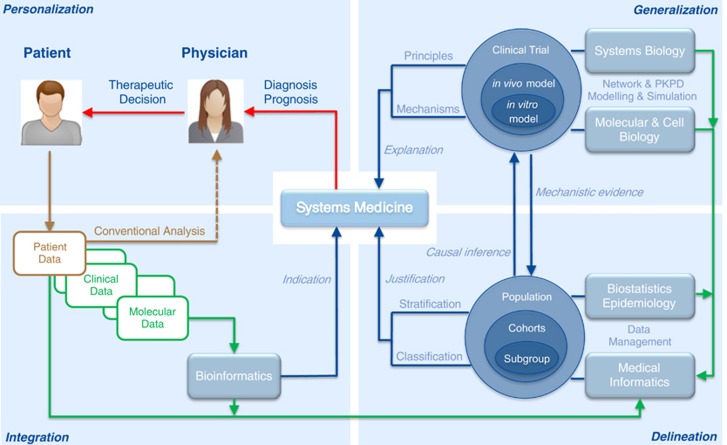
Systems medicine as an integrative approach, combining technologies, data, methodologies and expertise. Brown: Conventional analysis of patient data. Green: Data flow. Blue: Information flow, linked to the disciplinary expertise involved.

When it comes to defining the factors that are needed to understand a system, this will typically encompass heterogeneous data sources, including a wide range of technologies to generate new data and retrieve archived data. The integration of data, ranging from patient records to sequencing and multi-omics technologies, as well as databases from external data sources such as drug targets, molecular pathways or clinical trials (Figure 1), is a challenge that requires coordinated action at different levels.^15, 16, 17^ The IT infrastructure to manage such data (storage, provenance, security, sharing, user interfaces and process integration) is an important element to connect research and health care provided to patients. The analysis and interpretation of data requires the development of ‘integrative workflows’, combining multiple statistical, computational and mathematical techniques in a rational and reproducible process that can be implemented in software tools.^16^ While many tools already exist, the construction of workflows, the choice of appropriate methods and their adaptation to the medical context, should be more stringent and less of the intuitive and often ad hoc art form it currently is.

A systems approach holds great promise not only for clinical medicine but also for further clinical applications, for example, drug development.^18, 19^ Before identification of non-responders on the one hand, and a mechanistic understanding of the occurrence of adverse events on the other, will help to design targeted therapies. The interpretation of clinical information in systems pharmacology will ultimately challenge the ‘one-size-fits-all’ paradigm in pharmacotherapy and has the potential to support the accomplishment of personalized therapy designs with optimal risk-benefit ratios.

## Data availability, accountability, quality, analysis, integration and interpretation

The more data sources are connected to a patient, the more data provenance is of key interest. Not all data sources can provide the highest data quality. There is always a trade off on how much effort (and time) can be put into capturing data and how high the data quality has to fulfill its purpose. For example, clinical documentation, primarily used for capturing the rationale of a certain treatment, might be less relevant for research than reports from clinical trials. Likewise, data collected from electronic patient records will inevitably contain a lot of ‘noise’ and have to be cleaned before being used for research purposes.

Data provenance embraces high quality data sources. Standards for data provenance like ‘W3C Prov’ (https://www.w3.org/TR/prov-overview/) can be very helpful, but are rarely used by the biomedical community yet. There is already considerable investment into IT solutions for improved data provenance. However, medical informatics, without a systems medicine approach, is like building a house without the installation of switches to turn the light on. The need of data analysis, integration and interpretation, as well as the construction of reproducible workflows and validated methodologies, is increasing with data diversity, generated by an expanding arsenal of technologies. There still is a big gap in the availability of methods and software tools to perform such sophisticated analysis. Methods that allow analysis of high-dimensional data sets and multi-scale data integration have to be developed. Likewise, benchmark data sets need to be developed and made available in open source software.

## ‘Macroscopes’ to explain principles of tissue organization

One goal of systems medicine is to explain the emergence and progression of disease phenotypes with the help of molecular, cellular, physiological and environmental data. We are dealing with a multilevel and multiscale system. Diseases occur across a wide range of interlinked temporal and spatial scales (from the seconds and minutes of molecular reactions to the weeks and years during which diseases progress). By focusing on well-defined clinical questions, it is possible to develop context-specific models, which are not generic, but nevertheless predictive. In ecology, physics, meteorology and engineering we already rely entirely on predictive models for decision making and understanding of underlying causal mechanisms. Despite the challenges posed by biological complexity, advances in high-throughput technologies and data integration provide tremendous opportunities for data-driven modeling, which have yet to be realized.^20, 21^ To understand the emergence, progression and prevention of diseases, we must make inferences across multiple levels of structural and functional organization (for example, from molecules to cells and organs, from molecular reactions to tissue physiology, from molecules to MRI scans). How this can be achieved in a rational and practical way, remains an open scientific challenge.

Technologies generating detailed data about molecules and cells are our ‘microscopes’ that allow us to ‘zoom in’ and to study diseases at a molecular and cellular level. However, a reverse process is needed for bridging molecular and organ scales, taking us from molecular reactions to organ physiology. The process of ‘zooming out’, which is currently not supported by existing methodologies, requires the development of ‘macroscopes’ and a corresponding methodology by which we can abstract from cellular mechanisms to principles of tissue organization. The ‘macroscope’ provides a new paradigm for multiscale mathematical modeling and simulation. The development of ‘macroscopes’ also requires new experimental organ models, like artificial organs, and involves imaging techniques that support the integration of heterogeneous data across multiple levels of structural and functional organization in tissues and organs. Appropriate scaling, abstraction and generalization should allow a rational integration of evidence across multiple levels of time and space, linking clinical evidence with molecular data. The development of ‘macroscopes’ could then bridge the gap between basic research in molecular and cell biology, and clinical/physiological observations.

## Quantitative and single-cell technologies

Because most disease-related processes occur in space and time, their analysis requires quantitative time-resolved data. Despite recent progress in systems biology, one should not forget that virtually all omics-related technologies have still to realize their full potential in biomedicine. In addition, today we measure mostly summated signals from cell populations, which are, heterogeneous with respect to their activation kinetics. On the other hand, single-cell technologies, for example, single-cell sequencing and flow cytometry, support drug discovery and development and are improving our understanding of pathological processes.^22, 23^ There is an urgent need for advancing these technologies further and for developing new analysis algorithms. Single-cell quantitative techniques, including imaging flow cytometry, single-cell RNAseq, as well as multiplexed imaging-based single-cell analyses within tissue contexts generate large quantities of data and provide enormous challenges for data management, analysis and interpretation due to the heterogeneity of data types and the provenance of such datasets.^24^ Deep learning techniques that extract feature representations directly from pixel intensity values are making an impact in the field of computational biology. In particular, deep convolutional neural networks have brought about breakthroughs in processing images.^25^ Imaging flow cytometry, with only one cell per image, serves as an example for the application of deep learning and hence the need to develop this methodology for clinical applications. More generally, we see advances from machine learning and other computer science methods providing new opportunities for data processing and analysis in the life sciences.

## Implications for the patient

Conventional clinical data can be complemented with omics and sequencing data and by data on symptomatology and quality-of-life generated by smartphones and smartwatches. At present, suitable tools are still missing to enable patients to gather data themselves and methodologies to support the analysis of such data. Initial assessments are often suffering from inaccurate information provided by patients from memory.^26^ Hence tools for standardized and secure data management are required. Beyond electronic health records, new technologies and methodologies could enable patients to contribute personal data for their own treatment and for research. With rules and standards executed without commercial bias and providing ample protection^27^ (see also FDA recommendations for mobile medical applications, https://www.fda.gov/downloads/MedicalDevices/DeviceRegulationandGuidance/GuidanceDocuments/UCM263366.pdf), patients would be more likely to contribute their data. These data might eventually save their and other people’s lives.

The processing power of smartphone chips is comparable to workstations a few years ago, so that even image processing and deep learning techniques to detect patterns in data sets can be envisaged. The new technologies and methodologies could put patients in the position to know, own and share their own health data. This would improve the accuracy of diagnosis and speed up personalized therapeutic decisions.

## Involvement of stakeholders

Systems medicine holds the promise of disease prevention and improved patient care. There are two critical steps for making it work. Additional support by relevant stakeholders is required. First, during the ‘investment phase’ (wherein clinical problems are defined, models are developed and data sets are generated), the clinical setting has to be suitable, which requires to dedicate specific units (‘systems clinics’). This includes, for example, flexible and interactive workflows of patient care, involvement of ethics committees, sufficient time for extensive patient examination, and last but not least, basic training and awareness of medical students in the molecular and mathematical foundations of systems medicine. Second, during the ‘harvesting phase’, health care system providers, health insurance companies and regulatory agencies, could get involved to realize the gain of systems medicine and translate it into clinical practice.

## Conclusions and outlook

Systems medicine is a multi-scale, multidisciplinary approach to medicine. There is no doubt that medicine is becoming data-rich. A major challenge is to combine and interpret the data. Whether medicine will become more personal, depends very much on our ability to interpret complex heterogeneous data sets. Whether medicine becomes more precise will largely depend on our ability to integrate data. Both tasks can only be realized by systems medicine approaches, as defined above. The research gaps, described above, provide opportunities but also reflect clinical needs. The need is for new methodologies but also for trained personnel. With medicine becoming data-driven and quantitative, it is vital to establish interdisciplinary teams at the interface of medicine and the physical and engineering sciences. The analysis of new data arising in medicine cannot be processed in a fully automated form and can also not only be provided or analyzed in a service manner. For the coming years, we require more data scientists, specialising on clinical and health data and we require more clinicians and data scientists who understand each other's scientific language. The best health care is provided by hospitals that also promote research. The boundaries that separate patient care and research need to be broken down. Systems medicine is such an interdisciplinary approach to understand disease mechanisms and/or improve the diagnosis, therapy and outcome of diseases through the integration of data from a wide range of sources and types. Would teams adopting a systems medicine approach not emerge naturally? Data are the basis for decisions in health care. Modern technologies complement patient data with genetic and molecular information. It is not so much the quantity but the heterogeneity of the data that creates challenges. This makes it impossible to analyze and interpret the data in a meaningful way without computational, statistical and mathematical tools. Given the complexity of human health and disease, only a multidisciplinary approach can help reduce uncertainty in analyses and improve the accuracy of predictions.

Multidisciplinary approaches are not unknown to medicine, ‘tumor boards’ and ‘transplantation boards’ are successful examples from current practice. Experience from biology tells us that in order to establish multidisciplinary teams, one has to explicitly support this. In molecular and cell biology, the use of computational and systems approaches was introduced about 20 years ago. What we consider today as normal, biologists working in interdisciplinary teams, did not come about without programmes to encourage and support the formation of these teams. Whereas in biology teams will typically consist of a limited number of partners, in systems medicine we are dealing with larger teams, involving primary care physicians, specialized clinicians and epidemiologists, working in teams with experts from biology, biostatistics, medical informatics, bioinformatics, mathematics and computational modeling. The practical experience of pilot projects adopting a systems medicine approach show us that research and health care currently do not mix well. Some of these problems are addressed with the implementation of new IT systems but there are also strong cultural barriers. The organization of clinical processes and career development provide a barrier for the engagement of physicians in research and for more quantitative and data-driven approaches. Given the scale and complexity of clinical processes, the introduction of changes in medicine will require more than just new technologies and tools for data analysis.

The business-oriented and finance-driven organization of clinics provides a barrier to establish an interdisciplinary institutional vision. The image of doctors using general purpose and commercial Internet search engines for analysis and decision-making is a worrying scenario. So, what if systems medicine does not become a reality? New technologies are already changing medicine but without new methodologies to analyze and interpret the data, this change will not be effective. Without systems medicine, electronic health records become data graveyards. The second aspect of the doctor-patient relationship, supported by systems medicine, is the link between the patient’s individual condition and information from other patients, cohorts and populations. IT systems provide the basis to integrate and analyze data as has never been possible before. However, it would be fatal to believe that collected data by itself will improve diagnostic and therapeutic strategies. We have to make the second step, that is, to develop and use (new) methods to integrate, analyze and interpret the data, that is, to apply systems medicine. The treatment of patients thereby becomes not only more data- (and evidence-) based but also more precise because the patient’s data can be compared to other cases and clinical practice guidelines and references. The analysis of such heterogeneous data sets is a major scientific challenge but with very high gain compared to the current practice. Without systems medicine, the opportunities that arise from the analysis of data will be lost.

Systems medicine is a novel multidisciplinary approach to medicine, realising a methodological pipeline consisting of quantitative technologies to generate data, information systems for data management and methodologies for data analysis and interpretation. There is clear potential for a high benefit of a systems medicine approach for patients. The key to its success is the formation of interdisciplinary teams and the integration of data. This requires training and education in quantitative approaches and decision-making and this should start in the medical school. The framework in which systems medicine operates should become an integral part of the medical curriculum.

Changing well established organizational structures is not easy. What we described as a way forward to address these challenges are new ‘ways of thinking’ about diseases and data. Although this change will not occur by itself and will require governmental action, we are optimistic about systems medicine providing real benefits to patients.
